# Enhancing general spatial skills of young visually impaired people with a programmable distance discrimination training: a case control study

**DOI:** 10.1186/s12984-019-0580-2

**Published:** 2019-08-28

**Authors:** Fabrizio Leo, Elisabetta Ferrari, Caterina Baccelliere, Juan Zarate, Herbert Shea, Elena Cocchi, Aleksander Waszkielewicz, Luca Brayda

**Affiliations:** 1 Robotics, Brain and Cognitive Sciences Department, Fondazione Istituto Italiano di Tecnologia, Genoa, Italy; 2 LMTS, Ecole Polytechnique Fédérale de Lausanne (EPFL), Neuchâtel, Switzerland; 3 Istituto David Chiossone, Genoa, Italy; 4 Fundacja Instytut Rozwoju Regionalnego, Krakow, Poland

**Keywords:** Visual impairment, Distance estimation, Training, Programmable tactile displays, Learning, Haptics

## Abstract

**Background:**

The estimation of relative distance is a perceptual task used extensively in everyday life. This important skill suffers from biases that may be more pronounced when estimation is based on haptics. This is especially true for the blind and visually impaired, for which haptic estimation of distances is paramount but not systematically trained. We investigated whether a programmable tactile display, used autonomously, can improve distance discrimination ability in blind and severely visually impaired youngsters between 7 and 22 years-old.

**Methods:**

Training consisted of four weekly sessions in which participants were asked to haptically find, on the programmable tactile display, the pairs of squares which were separated by the shortest and longest distance in tactile images with multiple squares. A battery of haptic tests with raised-line drawings was administered before and after training, and scores were compared to those of a control group that did only the haptic battery, without doing the distance discrimination training on the tactile display.

**Results:**

Both blind and severely impaired youngsters became more accurate and faster at the task during training. In haptic battery results, blind and severely impaired youngsters who used the programmable display improved in three and two tests, respectively. In contrast, in the control groups, the blind control group improved in only one test, and the severely visually impaired in no tests.

**Conclusions:**

Distance discrimination skills can be trained equally well in both blind and severely impaired participants. More importantly, autonomous training with the programmable tactile display had generalized effects beyond the trained task. Participants improved not only in the size discrimination test but also in memory span tests. Our study shows that tactile stimulation training that requires minimal human assistance can effectively improve generic spatial skills.

**Electronic supplementary material:**

The online version of this article (10.1186/s12984-019-0580-2) contains supplementary material, which is available to authorized users.

## Background

The estimation of distances is a perceptual task frequently employed in everyday life. It is used at school when measuring geometrical shapes and can be literally a life-saver if you consider a driver that needs to constantly estimate the distance between cars to avoid collisions. Although we take this ability for granted, from a neuroscientific point of view, this is a complex skill. For example, when using sight, estimating the distance between objects requires solving the potential confusion between object size and object distance. When using only haptic or kinesthetic information (e.g., as with blindness) to estimate distance, several possible biases emerge both in *manipulatory space*, a small-scale layout that can be explored with the arms, and *ambulatory space*, a larger scale space that must be explored on foot [1, 2]. In *manipulatory space*, [1] reported that the estimation of distance in sighted but blindfolded participants was distorted, but their estimation of angle for a triangular pathway was very accurate. The most common error is the overestimation of distance for short lengths and underestimation of distance for long lengths, called the *range effect* [3–7]. Other studies report an increasing overestimation of straight-line distance as the explored path length increases, the so-called *detour effect* [8, 9]. Furthermore, a line radially oriented relative to the body is judged longer than the same line tangentially orientated [10, 11]. Similar biases, albeit of reduced magnitude, have also been reported for the visual modality, suggesting a similar organization of spatial encoding in both domains [12–14].

Haptic distance estimation is influenced by task-specificity and response mode [15, 16]. For example, the relationship between physical length of a stimulus and its estimated size is linear with a slope ~ 1 when the estimation is performed using two index fingers, one at the beginning and one at the end of the stimulus [17], or with a single finger (or the whole hand) moved along the stimulus [18, 19]. However, the slope of the function increases to 1.2 if the estimate is given by the space between the index finger and the thumb in a pincer posture [17] and decreases to 0.885 when participants are asked to reproduce the stimulus length with the same index finger they used to explore [20]. Regardless of task design and response mode, greater inaccuracy in haptic distance estimation compared to visual distance estimation is a general finding of studies. Abundant evidence indicates that vision does much better on length perception tasks than use of haptics [21–24], although the role of the haptic modality can increase when haptic information is judged as more reliable in a specific context [25].

Investigating haptic distance perception in blind individuals is particularly important since this skill is crucial in this population for a wide range of tasks such as learning geometry, reading Braille, knowing the relative dimensions of objects used daily, and estimating distances in tactile maps and diagrams [26, 27]. Literature suggests that blind people often experience difficulties estimating distances. For example, [28] found that early blind participants make more mistakes when estimating distances compared to late blind participants in *manipulatory space*. A similar finding was observed in visually impaired children; [27] showed that blind and severely impaired children made more errors than sighted children when judging distances in *ambulatory space* after they explored a tactile map depicting that space. Other studies, however, reported similar performance in blind and sighted individuals both in *manipulatory* and *ambulatory space* [29, 30]. While the degree of visual disability may modulate distance estimation skills differently in different tasks, it remains clear that haptic distance estimation is less precise and reliable than its visual counterpart. Hence, visually impaired persons might benefit from specific training in this skill.

Indeed, there is a growing interest on finding novel training schemes where visually impaired persons can refine their spatial skills in partial autonomy: acoustic cues on surfaces with built in sensors have been shown to reduce haptic localization errors in blind participants [31]. Similarly, spatial training leads to learning effects in blind people performing shape-recognition or navigation tasks [32–36]. A recent review from the US Department of Education [37] reports that practitioners consider tactile devices the most suitable for conveying geometrical and mathematical concepts. Yet, research investigating effective intervention strategies is woefully absent [37] despite documented lower achievements in mathematics and geometry for visually-impaired students. Current solutions are devices that operate in the *manipulatory space*, including the Cubarithm slate, Braille, stick-on number lines, and raised-line drawings, cannot be updated or tailored to user needs without an external intervention that blind children rarely have. Practically, to the best of our knowledge, a system that allows autonomous training of tactospatial abilities in blind persons does not exist.

Here, we investigate whether distance discrimination ability in *manipulatory space* can be trained in visually impaired participants using a programmable tactile display. We designed a training methodologically similar to that used in [38], but with a different task and a different goal. We expect that the performance of blind and visually impaired young people may improve during the training. We also investigate whether basic distance discrimination ability is influenced by the degree of visual disability.

Our goal is different than [38]; we investigate not only if spatial ability significantly increases with training using a programmable tactile display, but also whether performance improvement in distance discrimination might generalize to other spatial skills. This is not a trivial issue as previous research provides conflicting evidence regarding the possibility of generalization in spatial tasks. For example, [39] found that expert Tetris players outperformed non-skilled players in mental rotation tasks involving figures similar to Tetris shapes but not in other spatial skills. Another study found that improvement in a spatial skill can generalize to another task of the same type [40]. However, a recent meta-analysis of 217 spatial training studies highlights evidence of improved spatial skills not directly trained [41]. The transfer of skills seems more likely when the two tasks rely on the same cognitive and/or motor process (e.g., [42]). The vast majority of studies investigating the transfer of spatial training skills focus on the visual modality. Much less is known about the haptic modality, particularly in persons who are blind. There is evidence that playing video games might elicit transfer of navigation and spatial cognition skills in the blind (e.g., [43]) but less is known about the transfer of skills in the *manipulatory space.*

To investigate whether such learning generalizes to other spatial skills in the *manipulatory space* in the blind, we administered a standard haptic test battery using raised-line drawings, both before and after training with the programmable tactile display. The haptic battery was designed to measure a wide range of tactile skills in visually impaired and sighted children and adolescents [44]. Haptic battery scores of those given training were compared to the scores of a control group who performed the haptic battery tests without doing the distance discrimination training. We hypothesize that we might observe higher scores in the post-test of the haptic battery in the experimental group and not in the control group. Particularly, if the transfer of the training requires that the two tasks are of the same type, we might expect higher scores only in the size discrimination test which is the test that most resembles the distance discrimination task used in training. Alternatively, skill transfer requires less specificity, e.g. the two tasks although different are not entirely independent as they share some cognitive or motor process, we might observe higher scores also in non-trained spatial tasks.

In summary, in this study we ask following research questions:

1. Does distance discrimination ability improve in visually impaired persons doing a distance discrimination training using a programmable tactile display?
2. Is this skill modulated by the degree of visual ability?
3. Does the learning acquired transfer to other tasks of the same or different type?

## Methods

### Participants

A group of 23 blind (BLI) and a group of 24 severely visually impaired youngsters (SVI) were recruited in part by the FIRR Foundation of Krakow, Poland, and in part by the Istituto David Chiossone, Genoa. All participants were naïve to the experiments and none had a cognitive impairment that could influence performance in the task. Each group was divided into an experimental group (EXP, *n* = 24) and a control group (CTR, *n* = 23). BLI EXP age range was 8–22 years (mean: 15.3; 5 females). BLI CTR age range was 8–22 years (mean: 14.3; 11 females). SVI EXP age range was 12–19 years (mean: 15.6; 8 females). SVI CTR age range was 7–18 years (mean: 13.9; 3 females). The EXP groups and some of the controls were tested at FIRR Foundation, while Chiossone hosted part of CTR groups testing. The participants’ families gave informed consent in compliance with the Declaration of Helsinki. The experimental protocol was approved by the local ethics committees.

### Materials and procedure

#### Pre- and post-tests: the Haptic-2D battery

**Fig. 1:**
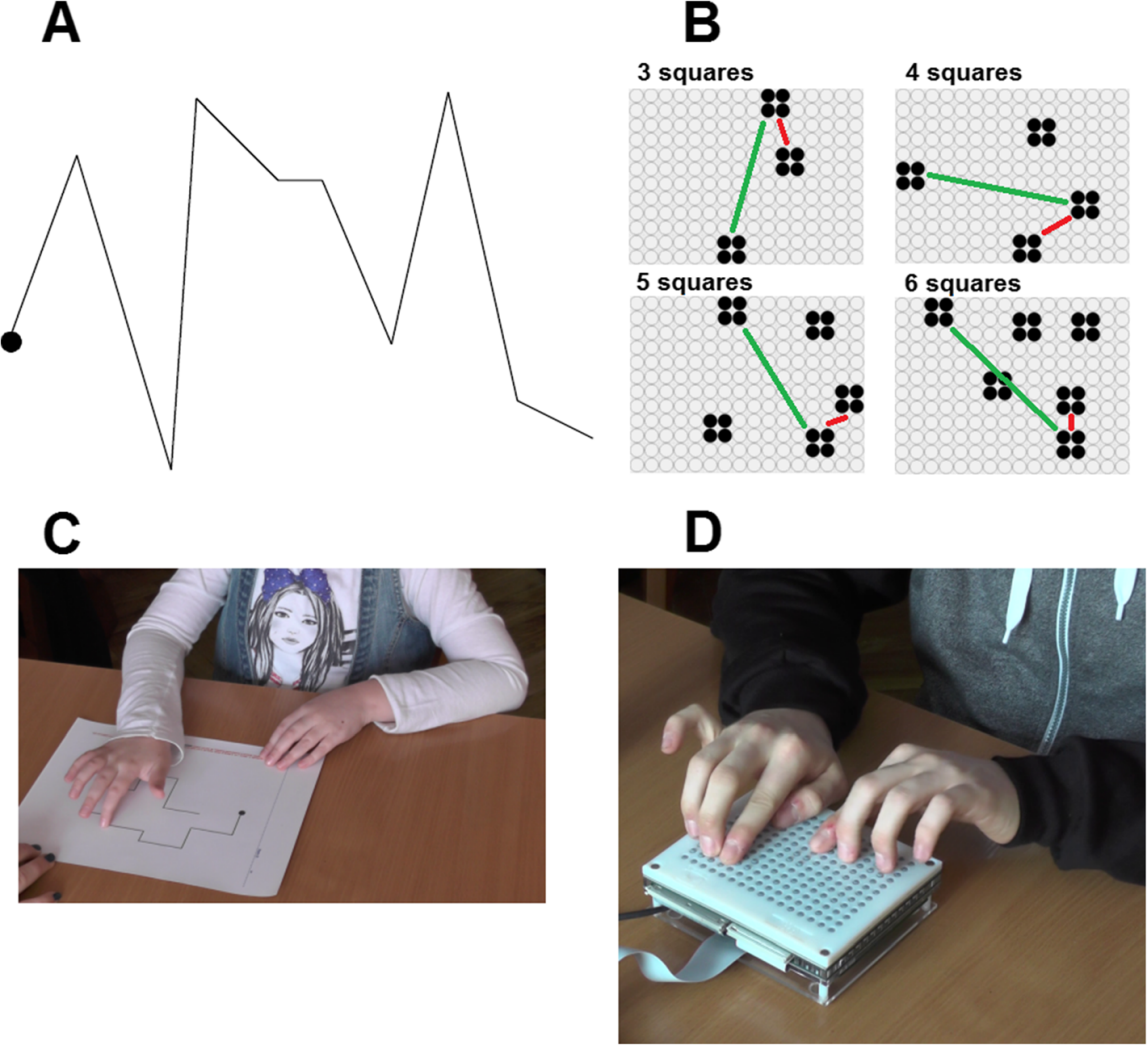
**a**. Example of a trial of the line scanning test of the Haptic-2D test battery. **b**. Examples of tactile images used in the distance discrimination training with a programmable tactile display with different number of squares drawn at pseudorandom locations. Red and green lines indicate the minimum (dmin) and the maximum (dmax) distance between squares, respectively. **c**. Child performing the line scanning test of the Haptic-2D test battery. **d**. Adolescent performing the distance discrimination training with the programmable tactile display

All participants were asked to perform a standard battery of haptic tests, the Haptic-2D [44]. This battery assesses the tactual abilities of sighted and visually impaired children and adolescents with two-dimensional raised-lines on size A4 sheets of paper. In particular, the battery measures five domains: scanning skills, tactile discrimination skills, spatial comprehension skills, short-term tactile memory, and comprehension of tactile pictures. More precisely, it is composed of 11 tests: dot scanning; line scanning; texture discrimination; shape discrimination; size discrimination; spatial location; spatial orientation; dot span; shape span; picture identification; picture completion (see [44] for a detailed description of the battery). The selection of these tests was based on the need to measure the haptic processing of 2D raised material that develops concomitantly with improvements in scanning, discrimination, spatial processing and short-term memorization skills [44]. Figure 1a and c show an example trial of the line scanning test and a blind child performing this test, respectively.

**Fig. 2:**
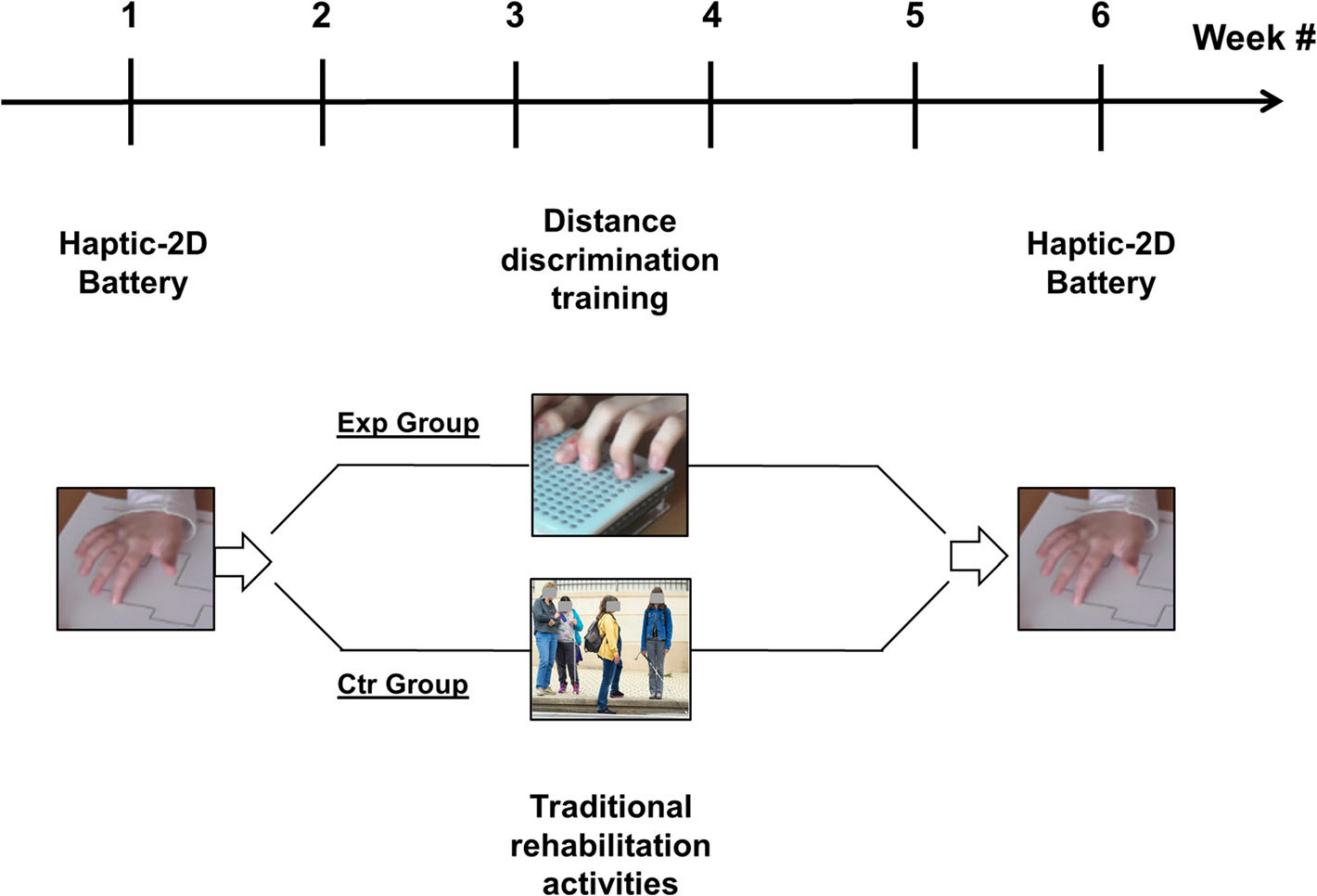
A schematic timeline of the experimental design. The activities of the EXP and CTR groups differed between the 2nd and the 5th week, with the former completing the distance discrimination training while the latter did only traditional rehabilitation activities

All participants were asked to do the battery twice (pre- and post-test) at a 5-week interval (see Fig. 2 for the study timeline). The EXP group performed the training with BlindPAD for one hour per week and did conventional rehabilitation practices in the remaining time. In particular, they completed a 4-week longitudinal training in a distance discrimination task using a programmable tactile display between the Haptic-2D pre- and post-test. Youngsters in the CTR groups did the pre- and post-test Haptic-2D battery, but instead of training with the programmable tactile display, these participants dedicated all their time, including the hour spent for the training in the EXP group, to conventional rehabilitation practices. Participants with some residual sight were blindfolded to avoid visual inspection of the materials.

#### Training apparatus: the BlindPAD

The BlindPAD is a refreshable array display of pins that move vertically [45]. Since the purpose of the array is to display tactile graphics, each pin is a tactile equivalent of a pixel and therefore called a ‘taxel.’ Underneath each taxel is a compact bi-stable electromagnetic actuator that pushes the pins up or down by 0.8 mm. Each taxel is individually addressed and can be set to be in the “up” or “down” state in under 20 ms. The row/column addressing architecture allows refreshing the entire display in under 2 s.

**Fig. 3:**
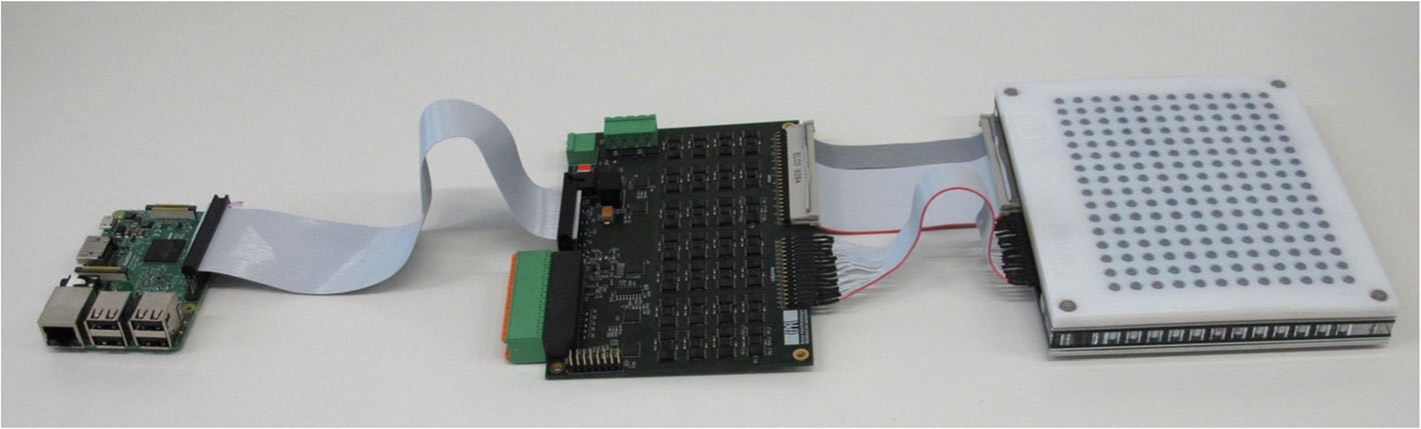
12 × 16 BlindPAD display (right) consisting of array of 12 × 16 latching electromagnetic actuators and a 3D printed pin interface, the control board (center) and a Raspberry Pi single-board computer (left). The control board is generally mounted under the display, as in Fig. 1. All 192 pins can be reconfigured in less than 2 s. Distance between pins is 8 mm and stroke is 0.8 mm

The BlindPAD display consists of a 12 × 16 array of actuators, an associated array of moving plastic taxels (the matrix of 192 grey dots on the right side of Fig. 3), and an electronic control board (center of Fig. 3) driven by a Raspberry Pi® single-board computer. The control board can be mounted under the display for a more compact arrangement. The desired patterns are generated on the computer (details are given in the next section) and transmitted to the display by USB connection to the Raspberry Pi.

**Fig. 4:**
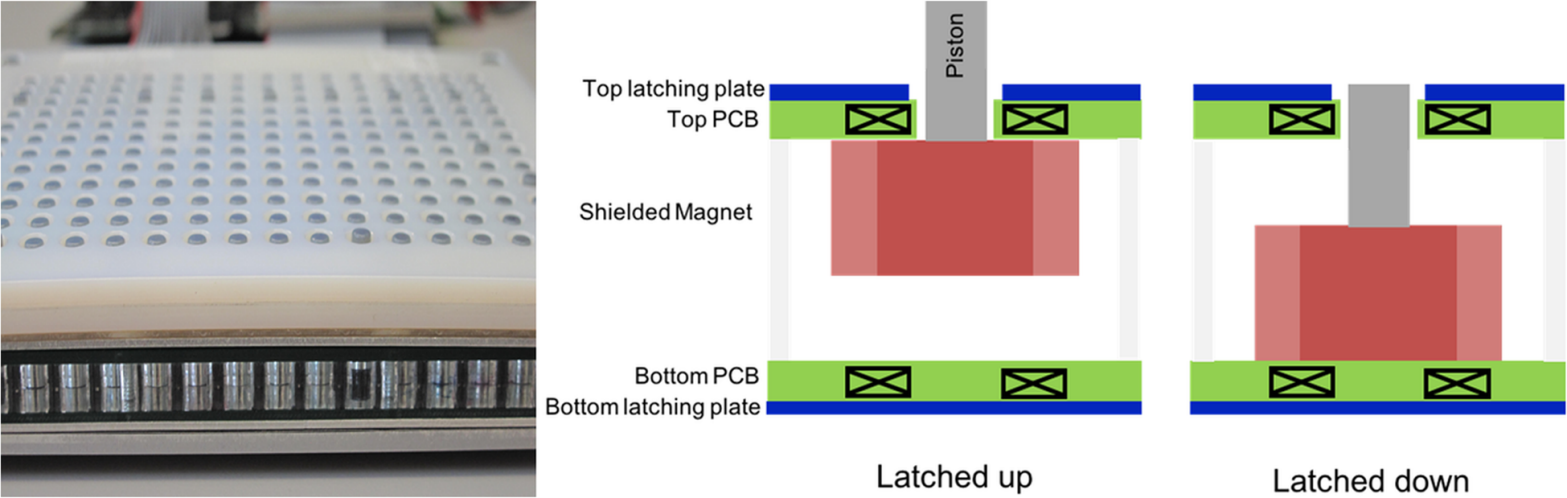
Left) Side view showing magnets (all are in down state except the fifth from the right), and 3D-printed pin interface (white with grey pins protruding). Right) schematic cross-section of one taxel, showing the two latched positons. To switch between the two stable positions, a current pulse is sent to the planar metal coils in both top and bottom printed circuit boards, generating a magnetic field gradient that pulls or pushes the magnet to the other stable state. The blue latching plates are ferromagnetic and hold the magnet in either the up or down state with no power consumption

Key challenges in dense arrays of electromagnetic actuators, such as that used here, are power consumption, cross-talk, force and displacement. The operating principle of the actuators is summarized in Fig. 4. Each electromagnetic actuator consists of a laterally shielded 6 mm diameter magnet that can slide up and down between two printed circuit boards (PCB). These PCBs contain 6-layer planar copper coils used to generate the magnetic field gradient that pull the magnet up or down. Above and below the PCBs are laser-cut sheets of soft iron, used to magnetically latch the magnet in either the up or down state. A central design consideration was scalability and compactness of the display: by using PCBs for the drive coils, rather than hand-wound coils, the display can readily be scaled to different sizes, and the overall thickness of the actuator layer is below 1 cm.

As the electromagnetic actuators are bi-stable, power is only required when switching states: the actuators can hold either an up or a down position indefinitely. Average power consumption per actuator pin is less than 100 mW, assuming a new image is displayed every 10 s, i.e., total average power consumption is under 20 W. Thanks to the low average power consumption, heating is also low, and is not perceptible by the user.

Cross-talk between taxels was eliminated by partially shielding each magnet to prevent magnets from exerting too strong a force on theirs neighbors, while still allowing efficient vertical actuation. Moving any taxel has no influence on all the other taxels. Since each taxel is individually addressed, a taxel can be vibrated at up to 10 Hz to draw attention to a specific pin.

The holding force is set by the magnet strength and the thickness and position of the latching plates. The average holding force is 200 mN, which is sufficient for most users. Latching force can be increased at the expense of higher power consumption (since more current is required to pull the magnet from the latching plate). We recently presented variants of this actuation technology in a non-latching configuration [46] and in the form of a flexible haptic strip for mobile applications [47].

The interface that the user touches transfers the force from the piston attached to the moving magnets (Fig. 4) to smooth plastic round pins of 4 mm diameter. Pins move up when driven by the magnet and drop back down when the magnet is pulled to the down state. The interface is 3D- printed on an Objet Connex printer, using two different colors of rigid VeroWhite material. The interface sets the vertical displacement of 0.8 mm. The taxel diameter and shape were chosen both for user comfort and for ease of pattern detection.

#### Training task: distance discrimination

For training, the BlindPAD’s Raspberry Pi is connected via wireless to a standard laptop and controlled by the software PadDraw, Matlab R2014 and Psychtoolbox 3.0.11 [48, 49]. PadDraw is a software developed by Geomobile GmbH within the scope of the FP7 EU BlindPAD project [50].

The two EXP groups (BLI and SVI) had four training sessions. Before the first training task, youngsters were familiarized with the tactile display. As in [38, 51], we adjusted the level of difficulty of the task for each participant at the beginning of each training session. We ran five trials for each level of difficulty, starting from the easiest, until the participant made at least one mistake. This level of difficulty was then used to start the training. Determining level of difficulty in this way allowed us to ensure the task was neither too easy nor too difficult, keeping the task challenging while preserving the possibility of performance improvements across sessions [38, 51].

Using Matlab, we prepared several sets of tactile images that included between three and six 2 × 2 taxel squares (see Fig. 1b for examples with different numbers of squares). This square is much larger than the single-taxel symbol we have shown already to be clearly perceivable by visually impaired users [52]. The four taxels of the square spanned a surface of 1.44 cm^2^, comparable to the contact area of a single fingertip under low contact forces [53]. The four taxels exerted together a maximum force on the fingertip of 0.8 N, leading to a pressure of 0.55 N/cm^2^. This is almost ten times the threshold (60 mN/cm^2^) to detect a dot on an otherwise smooth surface [54].

We used the same symbol (i.e., the 2 × 2 square) across the whole experiment, to avoid possible biases linked to recognition of different symbols. The same symbol, with equal inter-taxel distance (8 mm), was used in [46] and maximized recognition rate. The location of squares was pseudorandomly generated with one constraint: the minimum gap between squares was one taxel (i.e., two squares could not overlap or be continuous) to avoid confusion between possibly adjacent symbols. For each number of squares (from 3 to 6) we prepared 5 sequences of 20 images each, for a total of 400 different tactile images.

#### Procedure

At the beginning of the experiment, all the participants completed a Haptic-2D battery test (pre-test). Then, the CTR group completed standard rehabilitation activities (i.e., orientation and mobility exercises, psychomotor and social tasks related to visual rehabilitation) for 4 weeks while the EXP group performed a training session for one hour and did rehabilitation activities in the remaining time. In particular, the EXP group underwent a familiarization with the tactile display followed by four weekly training sessions. In each training session, participants completed 20 trials in which they were presented with an image such as the one shown in Fig. 1b. The participants were told to freely explore the surface of the device during the task. They had to judge which squares were separated by the shortest (dmin) and longest distance (dmax) (see panel B of Fig. 1). We manipulated the initial level of difficulty at the beginning of each session: the number of squares (from 3 to 6) was personalized according to each individual’s ability. We started with a 3-squares test, increasing the number of squares until the subject made at least one error. This procedure was repeated at the beginning of each session to set the difficulty level for that session. After the fourth session, all participants (EXP and CTR groups) repeated the Haptic-2D battery test (post-test).

#### Variables and statistical analyses

In the Haptic-2D battery, the dependent variable was the score of each test (to a maximum of 12 points per test, 132 points for the battery). Given the distance discrimination training received, we hypothesized that the mean score of the EXP group in both BLI and SVI participants would be higher in the post-test compared to the pre-test, at least in the size discrimination test of the Haptic-2D battery. In contrast, we expected that the scores of the two CTR groups should remain similar in the pre- and post-test. We further hypothesized higher scores in the SVI groups compared to BLI groups in the picture tests, which requires recognition of real-life objects (i.e., picture identification and picture completion tests). We expected that this could be due to higher recognition skills, associated with prior or superior visual experience.

In the distance discrimination training (performed only by BLI EXP and SVI EXP groups), three dependent variables were measured for each session: the level of difficulty reached, the response accuracy and mean response time (RT). Each variable was measured for both dmin and dmax.

The level of difficulty attained is expressed as the number of squares used during the training; in principle, the more squares on a tactile image, the greater the number of comparisons required to determine which pair is closest and which pair is furthest apart.

Response accuracy is defined as the ratio of number of correct answers to the total number of trials. This is raw accuracy. We also considered normalized accuracy in which raw accuracies from the second session onward were converted to performance differences (in percent) relative to the first session as the baseline. In this way, we were able to cumulate the relative improvements of the tasks both when difficulty levels remained the same across trials and when they changed [38].

Response time was measured as the time, in seconds, from the appearance of a tactile image (the BlindPAD allows an arbitrary number of taxels to be raised or lowered at precisely given times) to the time a participant indicates, with one or more fingers, the pair of squares (s) he thinks is closest and farthest.

We expected level of difficulty would increase during the training in both groups because of learning. Similarly, we expected response accuracy to improve during the training. Based on our previous studies using programmable tactile displays, we also might expect a trend toward greater accuracy enhancement in the SVI compared to the BLI group. For the response times, we expected faster RT at the end of the training compared to the beginning. BLI might be also faster than SVI since they are more familiar with haptic-only exploration.

Our independent variables were the group (CTR vs EXP), the degree of visual impairment (BLI vs SVI), the number of the training session within the training, and time (pre- or post-test).

Whenever data were not normally distributed, as indicated by Shapiro-Wilk tests, we employed non-parametric statistics. Within-group statistics were performed using Friedman ANOVAs followed by Wilcoxon signed-rank tests post hoc. All between-group differences were evaluated using Kruskal-Wallis tests followed by Mann-Whitney U tests post hoc.

Statistical significance was set at *p* < 0.05. Correction for multiple comparisons, when necessary, was conducted using the False Discovery Rate (FDR) control based on the Benjamini-Hochberg methods [55, 56].

## Results

In the following subsections, we first report the results of the Haptic-2D battery tests. Then, for the distance discrimination training, we report the level of difficulty, response accuracy (normalized data first, then raw data) and response time results.

### Haptic-2D battery

We investigated the effect of time (pre- vs post-test), visual disability (BLI vs SVI) and Group (EXP vs CTR) on the Haptic-2D battery scores using a 3-way mixed-model ANOVA, followed by post-hoc Scheffe’s tests (see also Table 1).

**Table 1:**
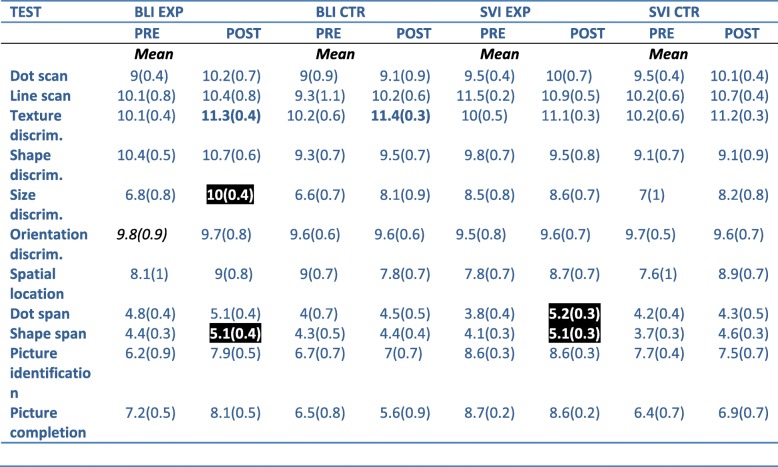
Mean scores on the 2D-Haptic test battery for BLI EXP, BLI CTR, SVI EXP and SVI CTR groups

Standard errors of the mean are reported in parentheses. Higher values indicate better performance; for each test maximum score is 12. Significant improvements in the post-test relative to the pre-test are indicated in bold. In particular, while the improvements shown in black bold are evident in both CTR and EXP group and are therefore unlinked to the distance discrimination training, the improvements in white bold on black background are exclusively obtained after the training. These skills (Shape span and Size discrimination in BLIND, Shape span and Dot discrimination in SVI) are the generalized spatial skills improved thanks to the distance discrimination training

**Fig. 5:**
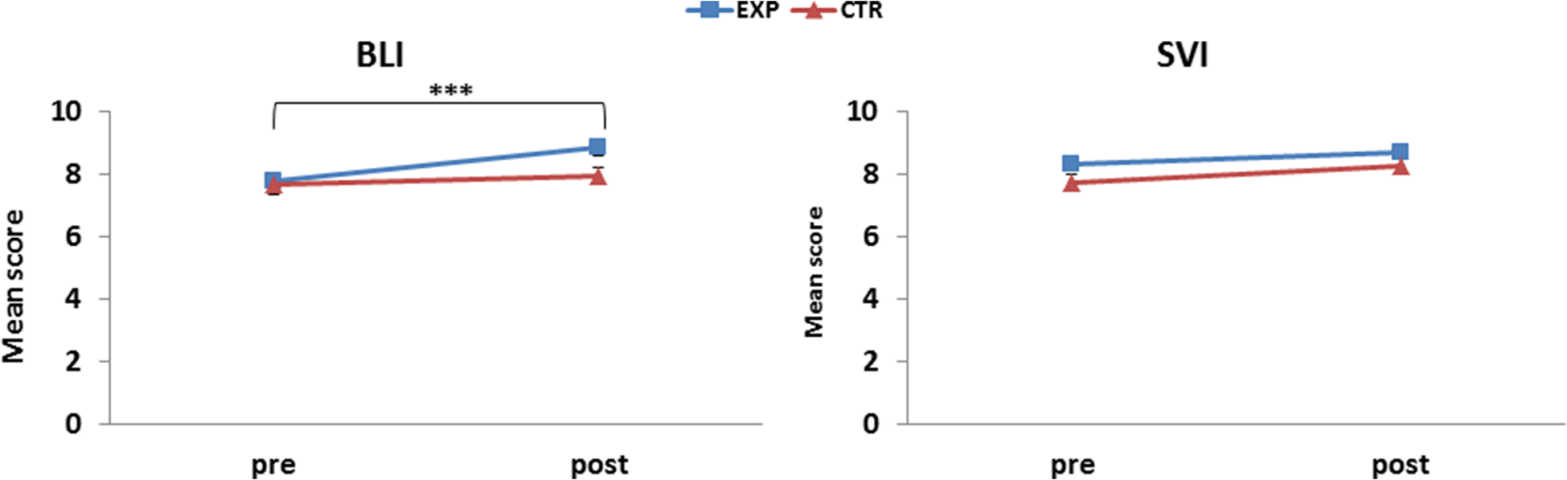
Left panel. Mean haptic-2D battery scores (all tests averaged) in the BLI EXP and BLI CTR groups. Right panel. Mean haptic-2D battery scores in the SVI EXP and SVI CTR groups. SEM are indicated as whiskers. Asterisks indicate a significant difference between scores in the pre- vs. post-test in the BLI EXP. ***, *p* < 0.001

The factor Group significantly affected (*F*_1,497_ = 4.36, *p =* 0.03) score, with the EXP group obtaining higher scores than the CTR group (8.42 vs. 7.92, *p* = 0.04). We also found an effect of the factor Time (*F*_1,497_ = 37.36, *p <* 0.001). The scores in the post-tests were significantly higher than scores in the pre-test (8.47 vs. 7.9, *p* < 0.001). The ANOVA revealed a significant 3-way interaction for Time x Visual disability x Group (*F*_1,497_ = 5.87, *p =* 0.015), resulting from only BLI EXP performance being significantly higher in the post- than the pre-test (8.85 vs. 7.8, *p* < 0.001; Fig. 5). All the other comparisons, including the comparison between SVI EXP and CTR in the pre-test (*p* = .91), SVI CTR pre-test and post-test (*p* = .27), SVI EXP pre-test and post-test (*p* = .74) were not significant.

Since the previous analysis did not allow us to investigate separately the scores of the subtests, we also compared the pre- and post-test scores for each subtest using Wilcoxon signed-rank tests. Since this is an exploratory analysis involving eleven subtests, we report both the uncorrected and FDR corrected *p*-values.

For BLI EXP, post-test scores were higher than pre-test scores in the texture discrimination test (11.3 vs 10.1; Z = 2.25; *p* uncorrected = 0.02, *p*FDR-corrected = 0.13), size discrimination test (10 vs 6.8; Z = 2.62; *p* uncorrected = 0.008, *p*FDR-corrected = 0.088), and shape span (5.1 vs 4.4; Z = 2.03; *p* uncorrected = 0.04, *p*FDR-corrected = 0.14). In contrast, the BLI CTR group improved only in the texture discrimination test (11.4 vs 9.2; Z = 2.20; *p* uncorrected = 0.02, *p*FDR-corrected = 0.18). In the SVI EXP, post-test were higher than pre-test in the dot span (5.2 vs 3.8; Z = 2.35; *p* uncorrected = 0.018, *p*FDR-corrected = 0.099) and in the shape span test (5.1 vs 4.1; Z = 2.52; *p* uncorrected = 0.011, *p*FDR-corrected = 0.099). The SVI CTR group post-test scores did not differ significantly from pre-test scores.

These results show that the distance discrimination training effect transfers not only to tasks of the same type (i.e., size discrimination) but also to different spatial skills (e.g. shape and dot span).

Following our hypotheses, we also checked whether the SVI scores in the pre- and post-test were higher than BLI scores, at least for the picture tests in which recognition of real-life objects is required. SVI score was higher in the picture identification pre-test (mean BLI: 6.8, mean SVI: 8.1; U = 141, *p*FDR-corrected = 0.006), but not in the post-test (*p* > 0.09).

Finally, we investigated whether the age of participants modulates the scores of the Haptic-2D battery. To do so, we merged BLI and SVI as well as EXP and CTR groups and computed Spearman correlations between age and both pre- and post-test scores for the tests that were affected by the training (i.e. texture discrimination, size discrimination, shape span, dot span). Age correlated positively with score in the size discrimination post-test (*r*_*s*_ = 0.42, *p* uncorrected = 0.003, *p*FDR-corrected = 0.006) and tended to correlate positively with score in the shape span post-test (*r*_*s*_ = 0.36, *p* uncorrected = 0.014, *p*FDR-corrected = 0.056). The significant correlation between age and scores in the size discrimination post-test seems to be mainly due to the fact that older BLI participants improved more in that test (*r*_*s*_ = 0.49, *p* uncorrected = 0.02, *p*FDR-corrected = 0.08). No correlation exists between age and score in the size discrimination post-test for the SVI group, nor is the correlation effect modulated by belonging to the EXP or CTR group (all *p*FDR-corrected > 0.13).

### Distance discrimination training

#### Level of difficulty

**Fig. 6:**
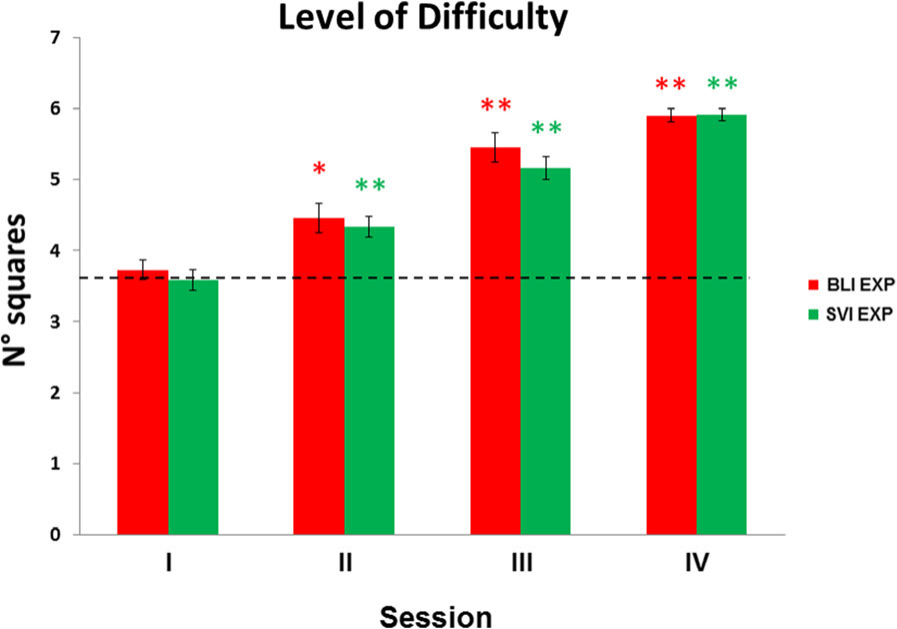
Number of squares used in BLI EXP and SVI EXP participants during the training. SEM are indicated as whiskers. Red and green asterisks indicate higher level of difficulty compared to the baseline in the BLI EXP and in the SVI EXP, respectively. Dashed black line represents the average number of squares at the baseline. *, *p*FDR-corrected < 0.05; **, *p*FDR-corrected < 0.01

Level of difficulty was expressed as the number of squares used during training. Number of squares used increased across sessions in both BLI and SVI groups as shown in Fig. 6.

Session significantly affected the number of squares used in the BLI group (Friedman ANOVA; *χ*^*2*^ = 28.51; *p* < 0.001). The number of squares used was significantly higher in sessions II to IV, compared to the baseline (all *p*FDR-corrected < 0.02; Fig. 6). Likewise, the number of squares used increased in sessions II to the IV compared to the baseline for the SVI group (*χ*^*2*^ = 32.88; *p* < 0.001; all post-hoc comparison *p*FDR-corrected < 0.01). BLI and SVI did not differ in mean number of squares used within a session (all *p* > 0.28). These results highlight that performance improvement due to the learning leads to an increase in the level of difficulty achieved by participants, as observed in [38, 51].

#### Response accuracy

We present the normalized data first followed by the raw data. Recall that the normalized accuracy data, in our paradigm, allow us to measure learning effects when level of difficulty changes [38, 51]. In contrast, the raw data give absolute values of accuracy regardless of the level of difficulty. Thus, the raw accuracy data cannot highlight some learning effects (e.g., the same absolute level of accuracy of a session with higher level of difficulty than the previous session).

##### Normalized accuracy

Accuracy in identifying dmin and dmax was statistically different in two of the four sessions; hence, we analyzed dmin and dmax data separately.

**Fig. 7:**
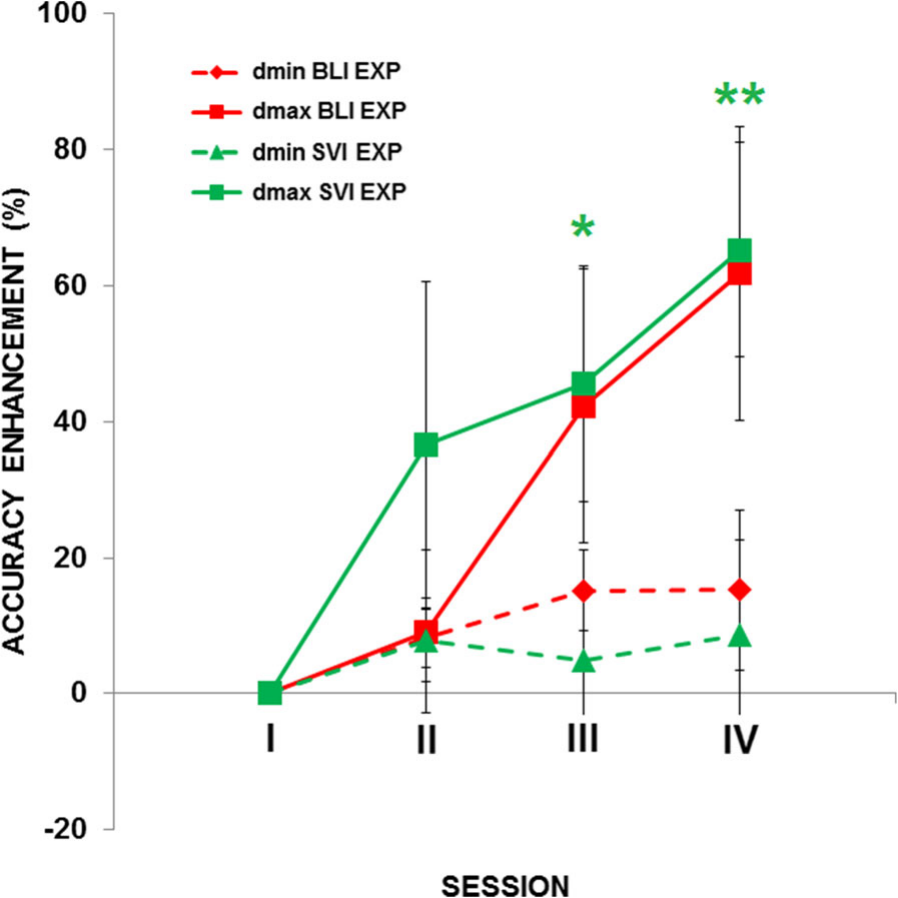
Normalized accuracy enhancement (SEM indicated as whiskers) across sessions in the distance discrimination training using the programmable tactile display. Color coded asterisks indicate higher accuracy compared to the baseline (Session I). *, *p*FDR-corrected < 0.05; **, *p*FDR-corrected < 0.01

BLI EXP and SVI EXP both had significant learning effects in the distance discrimination training (Fig. 7). Accuracy for dmax increased significantly in BLI (*χ*^*2*^ = 11.47; *p* = 0.009). In particular, session IV accuracy was significantly higher than baseline (Z = 2.31; *p* uncorrected = 0.02, *p*FDR-corrected = 0.06). Accuracy for dmax also increased in the SVI group (*χ*^*2*^ = 11.97; *p* = 0.007), with sessions III and IV having higher accuracy than baseline (both *p*FDR-corrected < 0.05). In contrast, for dmin only a marginal learning effect is present in BLI (*χ*^*2*^ = 6.43; *p* = 0.09) and no effect is present in SVI (*χ*^*2*^ = 2; *p* = 0.57). The difference in learning for dmin and dmax may reflect a ceiling effect. Accuracy for dmin at the baseline was ~ 88% in both BLI and SVI, while accuracy for dmax was ~ 66% in BLI and 60% in SVI. Learning effects did not differ between groups for dmin (all *p* > 0.35) or for dmax (all *p* > 0.87). Collectively, as observed for the level of difficulty, the normalized accuracy data show clear learning effects.

We also investigated whether age of participants and accuracy enhancement are correlated at the end of the training, separately for dmin and dmax. To do so, we merged the BLI and SVI groups. Age is not correlated with learning for dmin (*r*_*s*_ = − 0.11, *p* = 0.60) or dmax (*r*_*s*_ = − 0.23, *p* = 0.28).

Since the locations of the tactile squares were randomly generated, it is possible that the generated distances between squares were sometimes too similar to be discriminated (i.e., they were below the just-noticeable difference [JND] for distance discrimination). This would lead us to underestimate learning effects. To address this, we defined a tolerance response range (16.67%) based on previous results on length discrimination of raised lines (e.g., [57]) and revised the data to consider a subject’s response correct if it fell within this range. Results of the analysis were similar to the analyses presented above, confirming learning effects in both groups. The alternate analysis is reported in the Additional file 1.

##### Raw accuracy: number of correct responses

Firstly, we compared BLI and SVI in terms of raw accuracy for each session both for dmin and dmax. Raw accuracy of BLI and SVI groups was similar throughout the training for both dmin and dmax (all *p* > 0.09), suggesting that distance discrimination ability in this task is not affected by the level of visual impairment. Hence, we merged BLI and SVI data for the following raw accuracy analyses.

**Fig. 8:**
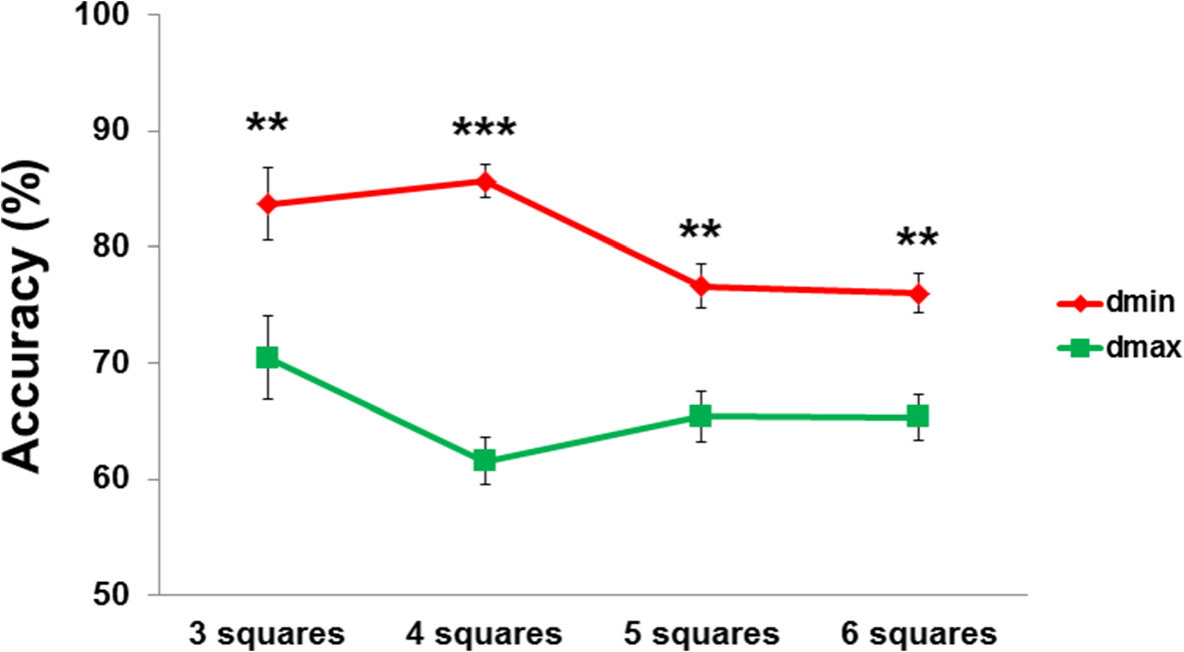
Response accuracy for each level of difficulty after averaging for BLI and SVI both for dmin and dmax. Whiskers represent SEM. Asterisks indicate higher accuracy for dmin compared to dmax. **, *p*FDR-corrected < 0.01; ***, *p*FDR-corrected < 0.001

Participants judged longer distances less accurately than shorter distances; 65% accuracy for dmax compared to 80% accuracy for dmin (Z = 8.70, *p* < 0.001; Fig. 8). This effect occurred consistently across all the levels of difficulty (all *p*FDR-corrected < 0.01) and might be due to different efficiencies in strategies used to discriminate shorter distances versus longer distances (e.g., counting the taxels might be efficient only for shorter distances).

We next investigated whether level of difficulty modulated response accuracy. Level of difficulty did not influence accuracy for dmin (χ^2^ = 3.06, *p* = 0.38) or dmax (χ^2^ = 1, *p* = 0.61), suggesting that the density of information does not affect performance at this spatial scale. We also investigated what kind of mistakes the participants did (see Additional file 1).

#### Response time (RT)

**Fig. 9:**
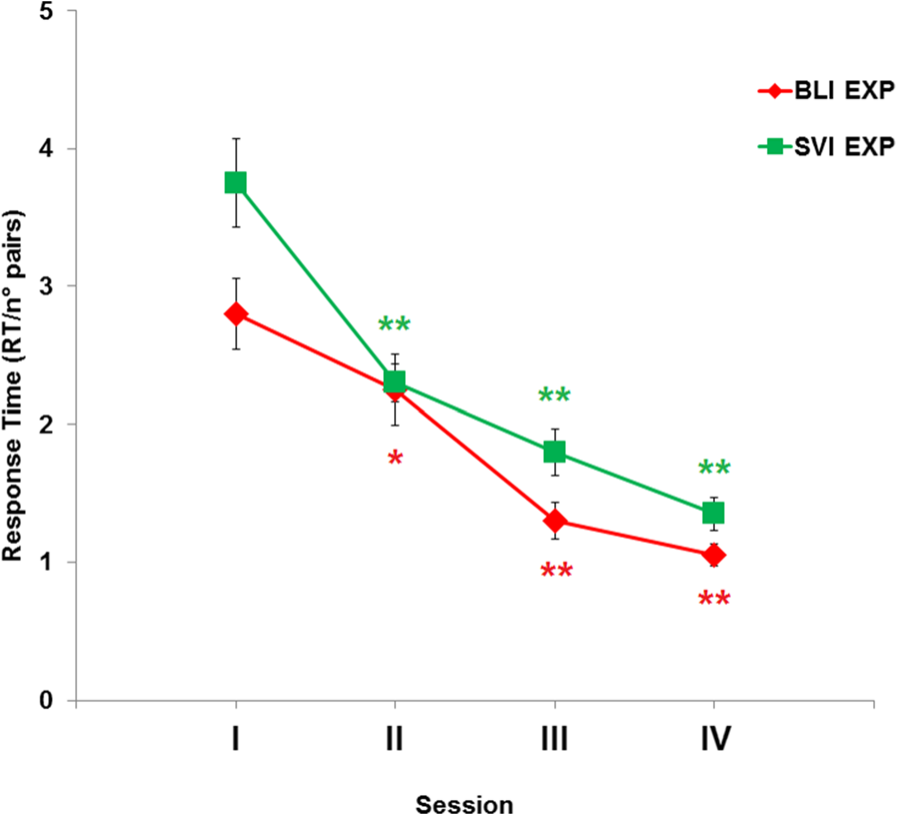
Normalized RT (SEM indicated as whiskers) across sessions in the distance discrimination task using the programmable tactile display. Color coded asterisks indicate faster RT compared to the baseline (Session I). *, *p*FDR-corrected < 0.05; **, *p*FDR-corrected < 0.01

RT notably slowed over the course of training, as we increased the number of squares. Thus, to assess the learning effects in RT, we normalized RT by dividing by the number of possible pairs in the tactile image (6 for 4 squares; 10 for 5 squares and 15 for 6 squares). Since response time for dmin and dmax were not statistically different for both BLI and SVI groups (BLI: 1.85 vs 1.90, Z = 0.03, *p* = 0.97; SVI: 2.22 vs 2.39, Z = 0.67, *p* = 0.50), dmin and dmax were averaged in the following analysis (Fig. 9).

Session number affected RT in both the BLI (*χ*^*2*^ = 27; *p* = 0.001) and SVI (*χ*^*2*^ = 30.7; *p* < 0.001) groups. RT decreased in sessions II to IV compared to the baseline in both groups (all *p*FDR-corrected < 0.05). Although RT appears to decrease more quickly in BLI compared to SVI (Fig. 9), response times were not statistically different between groups (all *p* > 0.05). Age did not correlate with RT in session IV (*r*_*s*_ = 0.09, *p* = 0.66). Overall, the faster response times observed at the end of training represent another piece of evidence, together with level of difficulty and accuracy, of a learning effect.

Finally, we attempted to find out whether the learning took place more at the intra-session or at the inter-session level (see Additional file 1). Briefly here, data are not conclusive regarding this point and further experiments will be necessary to answer this question.

## Discussion

This work represents, to the best of our knowledge, the first demonstration that a set of spatial abilities can be improved by means of a distance discrimination training, delivered with a refreshable tactile display, in visually impaired youngsters. Furthermore, in this work we show that:

1. both blind and severely visually impaired youngsters improve their distance discrimination ability in the *manipulatory* space during 4 weeks of training on a refreshable tactile display;
2. the amount of improvement does not depend on the level of visual impairment;
3. learning transfer occurs not only for abilities of the same type but also for non-trained tactile abilities.

We evaluated whether the ability to discriminate different distances is enhanced in visually impaired youngsters using a refreshable pin-array tactile display. Training this ability is particularly relevant for two main reasons. First, distance estimation skills are used in many spatial tasks, such as learning geometry, map reading, object discrimination and manipulation, and Braille reading. Despite of this, to our knowledge no specific standard instruments exist to train this skill, or size discrimination, in *manipulatory* space and just one test exists that assesses size discrimination ability in visually impaired children aged between 7 and 13 [58]. Second, haptic distance perception relies on encoding heuristics that might bias the perception itself (e.g., [28]). To verify whether distance discrimination can be trained, we designed a novel task in which participants had to find, in a 2D multi-squares tactile image, the two squares separated by the shortest and longest distance. Participants repeated the training task once a week for 4 weeks. The level of difficulty was matched to each participant’s ability by varying the number of squares that composed the tactile image. This allowed also to adapt the test to visually impaired persons of different ages. We found that both blind and severely visually impaired participants improved distance discrimination skills during the training. The level of difficulty reached at the end of the training was higher than the level at the beginning. More specifically, the mean accuracy (expressed as the percent of correctly identified shortest and longest distances among tactile symbols) increased relative to the baseline established in session I by 39 and 37% in blind and severely visually impaired participants, respectively, during the training. Furthermore, participants performed the task more quickly. Normalized RT (the time to judge each possible pair of squares) was also significantly faster than baseline in both the blind and the severely impaired (blind: 0.9 vs. 2.4 s; severely impaired: 1.2 vs. 3.3 s). This improvement might be due to the fact that participants got used to the stimuli, to the interface and also learned to do the discrimination task more effectively. There is no way to disentangle between all of these variables but we think this is a common issue in most learning paradigms using response times as dependent variable.

Degree of visual ability does not modulate distance discrimination skills. The level of difficulty reached did not differ between BLI and SVI individuals at the beginning or end of training. Blind and SVI participants showed very similar performance enhancement at the end of the training and both became faster at performing the task across the training. This result agrees with that of studies employing single-images training using programmable tactile displays in blind and SVI persons [38, 51] and with research showing similar abilities in blind and sighted participants learning or exploring tactile images [59, 60].

### Effects of general spatial skills

We hypothesized that enhancement of the ability in discriminating distances could transfer to a tactile task of the same type (i.e., size discrimination) administered with raised-line drawings instead of a BlindPAD. We further hypothesized that transfer may also occur for different tasks that share some processes (e.g., working memory load). We thus administered a standard Haptic test battery designed to assess general tactual abilities, including size discrimination. Practically, the test battery was performed before and after the training. The scores in the battery were compared to the scores of a control group that performed the pre and post-test battery without doing the distance discrimination training with the BlindPAD refreshable display. We hypothesize that similar results could have been obtained with other refreshable tactile displays available on the market, in that the fine tactual abilities (that might be influenced by different dot pitch or taxel width or stroke) resulted unrelated to our training; however, the display should guarantee a refresh rate not too lower than 1 Hz to allow sufficient switching of tactile graphics.

Importantly, the blind experimental group improved in three subtests of the Haptic-2D battery (texture discrimination, size discrimination, shape span) while the control group improved in only one test (texture discrimination).

### Improvements in size discrimination linked to our training

Size discrimination test on raised-line drawings appears to be a task of the same kind as the distance discrimination training on the refreshable display. In fact, both tasks involve estimating and comparing distances. They are however different in that the paper-based size discrimination task requires to place the fingers on edges of the same symbol (e.g. a raised square or circle), while the distance discrimination task requires to place the fingers at the very end of an ideal straight line (the distance) joining two separated small symbols (the 2 × 2 taxel squares). The underlying spatial skill is, however the same and can be identified with the known ‘enclosure’ tactile exploration strategy [61].

### Improvements in texture discrimination not linked to our training

Since we found an improvement in texture discrimination both in the experimental and in the control group, such improvement is not linked with the training nor it is related to the use of a refreshable display, as compared to standard rehabilitation techniques. This result appears to reinforce our previous findings because improving spatial skills related to estimation of distances has little to do with skills related to texture discrimination. In other words, distance discrimination training did not require texture discrimination abilities finer than in any other task. The taxel squares were large enough and taxels were distant enough, far beyond tactile discrimination thresholds that might have been trained, had we used small or barely perceivable symbols. Our study, in fact, did not target fine abilities.

### Improvements in shape span linked to training on greater distances

While the effect of the training in the size discrimination test was expected, the reason for its effect on the shape span test is less clear. It might be due to an increased ability to estimate the envelope (a concept tightly connected to that of shape) of the ensemble of tactile symbols. This hypothesis is supported by the greater enhancement in estimating a longer distance (dmax) over a shorter distance (dmin). In other words, participants that become more proficient at judging the two most distant points of a flat shape also become better at estimating the overall shape. This is in line with the known link between exploratory procedures that serve to estimate a shape by enclosure [61]. Alternatively, this effect might be due to memory improvement with training. The latter hypothesis is supported by the fact that SVI participants who did the training improved in two memory tests (shape span and dot span). Indeed, at the end of the training, most participants did the task with six squares, so they had to keep in working memory the information to compare up to fifteen pairs of distances, which can be considered also as a sort of memory training. Previous studies provide evidence that it is possible to train spatial working memory and spatial skills in the blind [34, 35, 38, 51]. Overall, our findings suggest that training transfer can occur to non-trained tactile tasks using different stimuli but sharing similar cognitive and motor processes with the trained task, as suggested by [42].

### Different enhancements associated with different visual deprivations

Unexpectedly, the SVI experimental group did not improve in the size discrimination test which should be the test more influenced by the distance discrimination training. This lack of enhancement in the size discrimination test might be due to a ceiling effect: while the score of blind participants in this test before training was 6.8, the same score in the SVI group was 8.5, which is closer to the maximum score of 12.

We note a non-significant trend toward higher scores in the post-test for control groups. This average performance enhancement (5.7%) is similar to the 6.4% enhancement observed in [44] and might be due either to a performance improvement between test and retest or to the fact that participants felt more confident with the battery at the retest [44]. SVI youngsters obtained higher scores than blind participants in the picture identification test in the pre-test, suggesting that visual experience facilitates the recognition of tactile drawings depicting real-life objects [62–64], but see [65] for a different finding.

### Role of the distance discrimination training within standard rehabilitation practices

Our results show that a distance discrimination training refines spatial skills, while there is no skill that improves exclusively in the control group. The activities that were performed by the control group were different than a distance discrimination training, but were centered around development of tacto-spatial abilities, that include but were not limited to:

- Tactual activities at the desk: recognizing objects of common use in the kitchen, classroom
- Orientation and Mobility sessions: walking in unknown indoor and outdoor spaces, following walls with touch or learning the haptic response of a white cane
- Informatic classroom: familiarizing tactile feedback of Braille bars, learning to interact with keyboards
- Protection techniques while walking
- Musicotherapy on the piano

Since our training was done for about one hour per week, both groups went on with regular rehabilitation activities (balanced across groups) in the other hours. That is, the above list of activities was not abandoned in participants in the experimental group, but was skipped only in the hour of the distance discrimination training.

The blind experimental and blind control group were in the same range of age, which was quite wide (8–22 years old). Therefore, the participants within the control group performed all or only part of the list of activities (while their fellows in the experimental group underwent the distance discrimination training) since the rehabilitation programs were tailored to age and spatial abilities of the single participant. We cared about balancing the overall spatial abilities across groups before performing the experiment, that is: if two participants presented similar spatial skills, they were randomly assigned to either the control or experimental group. Therefore our results should not be biased by different spatial skills across groups.

This does not contrast with our results, because we measured skill improvements rather than absolute skills. Instead, our findings are reinforced by the fact that the distance discrimination training improves size discrimination and shape span skills across all ages in blind participants (and more in older blind participants) and that the age factor contributes equally to the training scores in the experimental or in the control groups.

The fact that the two groups mainly differ by performing the distance discrimination training made us conclude that the observed improvement in the Haptic battery scores was due to our training, all the other things being, to the best of our knowledge, equal.

### Contributions beyond the state of the art

This study goes well beyond previous findings [38, 51]. First, the training implemented here used a different programmable tactile display (i.e., BlindPAD) than in [38]. Although a comparison between different displays is beyond the scope of this study, the results demonstrate that learning effects can be obtained using programmable tactile displays with a lower resolution (number of taxels). Since the cost for these displays is roughly proportional to the number of moving pins, this result is important as it means that more affordable devices can be sufficient for effective rehabilitation.

In comparison to [38, 51], here we show that learning effects are not task-specific but instead generalize to different spatial tasks, even when administered with different media (i.e., raised-line drawings). While evidence exists that the effects of training of spatial skills can be generalized to non-practiced spatial tasks [31, 66, 67], to the best of our knowledge this is the first demonstration of transfer of tactile spatial learning effects in visually impaired youngsters. A previous study [68] investigating generalizations in learning to recognize facial expressions of emotions presented as raised-line drawings to blindfolded sighted and blind adults found generalization occurred in sighted participants, but not in the small sample of blind participants. Furthermore, the skill transfer observed in the sighted was limited to the training task (i.e., after the training, participants got faster at recognizing emotions of faces not previously presented). Whether learning generalized to different spatial skills, such as recognition of drawings of real-life objects, was not investigated.

One might argue that the improvement of the experimental group occurred, at least partially, because the control group may not be doing an equivalent but unrelated task. On the contrary, the training presented here was inserted into a standard rehabilitation session that lasted the same amount of time for both groups. In other words, while the experimental group was using BlindPAD, the control group kept on doing rehabilitation practices that spanned various tasks (such as orientation and mobility exercises, psychomotor and social tasks related to visual rehabilitation).

The ability to discriminate between different distances is important in many spatial tasks and is essential for learning geometrical concepts, as well as basic orientation and mobility skills. In addition, the use of programmable tactile displays makes possible autonomous training sessions. This peculiarity can in principle save a great amount of time of the practitioner, who spends the majority of his/her time preparing the material for each single end user. The use of programmable stimulation sequences make the program repeatable and requires to be prepared only once. Potentially, the stimulation can be performed outside traditional rehabilitation centers and even at home. We show that the training and the evaluation of one part of spatial abilities can be done in partial autonomy. Refreshable displays can become a tool that allow *spatial homework* to be part of more traditional rehabilitation programs, e.g., when the practitioner is not available. Alternatively, the training can be followed by the practitioner and the kind of exercise on the display and the level of difficulty can be decided offline or online. Spatial training like that proposed in our study could, in principle, be implemented using more traditional methods such as embossed paper. However, the whole training procedure would become rather cumbersome. Four hundred sheets of paper would be necessary to replicate our design and the continuous assistance of an experimenter would be required to change the sheet at the end of each trial, and to manually record accuracy and response times. In our view, the methodology we propose might serve as a complementary training tool that will scale up well for a worldwide population more and more affected by visual impairment [69].

## Conclusions

In this work, we show that visually impaired youngsters improve in haptic distance discrimination ability following training. We also show that the learning effect transfers not only to tasks of the same type on different media, but also to non-trained tactile tasks, such as short-term memory tasks. In addition:

1. We designed a new method to train distance discrimination using 2D multi-square images;
2. We showed how the improvement in the blind is similar to that of severely visually impaired youngsters;
3. We used BlindPAD, a new portable, low-resolution, refreshable haptic display that allows display of arbitrary tactile graphics.

## Additional files

Additional file 1: This file contains three additional analyses and relative figures. The first analysis considered the possibility that certain square distances might be below the JND for distance discrimination when measuring the training normalized accuracy. The second analysis measured the kind of mistakes participants did during the training. The third analysis attempted to find out whether learning effects took place more at intra-session or inter-session level. (DOCX 454 kb)

## Data Availability

The datasets used and analyzed during the current study are available from the corresponding author on reasonable request.

## Abbreviations

BLI: Blind
CTR: Control group
dmax: Longest distance between squares in a multi-square tactile image
dmin: Shortest distance between squares in a multi-square tactile image
EXP: Experimental group
FDR: False Discovery Rate
RT: Response time
SEM: Standard error of the mean
SVI: Severely visually impaired
