# Supplementary material for: Enhancing general spatial skills of young visually impaired people with a programmable distance discrimination training: a case control study
Source: J Neuroeng Rehabil. 2019 Aug 28;16:108. doi: 10.1186/s12984-019-0580-2 (PMC6714081; doi:10.1186/s12984-019-0580-2)
Supplement: Supplementary file 1 — This file contains three additional analyses and relative figures. The first analysis considered the possibility that certain square distances might be below the JND for distance discrimination when measuring the training normalized accuracy. The second analysis measured the kind of mistakes participants did during the training. The third analysis attempted to find out whether learning effects took place more at intra-session or inter-session level. (DOCX 454 kb) [file 12984_2019_580_MOESM1_ESM.docx]

**Thresholded accuracy**

As explained in the main text, accuracy data for those trials in which generated distances were possibly below the just-noticeable difference for distance discrimination were treated accordingly (e.g. if a response was wrong but the chosen distance was close to the distance corresponding to the correct answer (within 16.67%), the response was considered as correct).

Then, as we did for the analysis reported in the main text, response accuracy data were normalized to the I session baseline, i.e., accuracies from the II session onward were converted to percentage performance differences relative to the first session baseline.

We firstly compared learning effects for dmin and dmax for each session from the II to the IV. dmin and dmax performances were not statistically different (all *p*s > .10). Hence, we averaged dmin and dmax for the following analysis.

Supplementary Figure 1 shows BLI EXP and SVI EXP learning effects in the distance discrimination task. The learning effect in the BLI was significant (*χ^2^* = 11.91; *p* = .007). Particularly, session IV accuracy was significantly higher compared to the baseline (Z = 2.66; *p* uncorrected = .007, *p*FDR-corrected = .02). The learning effect was significant also in the SVI (*χ^2^* = 12.05; *p* = .007). Accuracy in the III and IV session was indeed significantly higher than the baseline, although the effects did not survive after FDR correction (both *p*s uncorrected < .05, *p*FDR-corrected = .07 and .054).

As for the group differences, we could not observe any effect (all *p*s > .62).

**
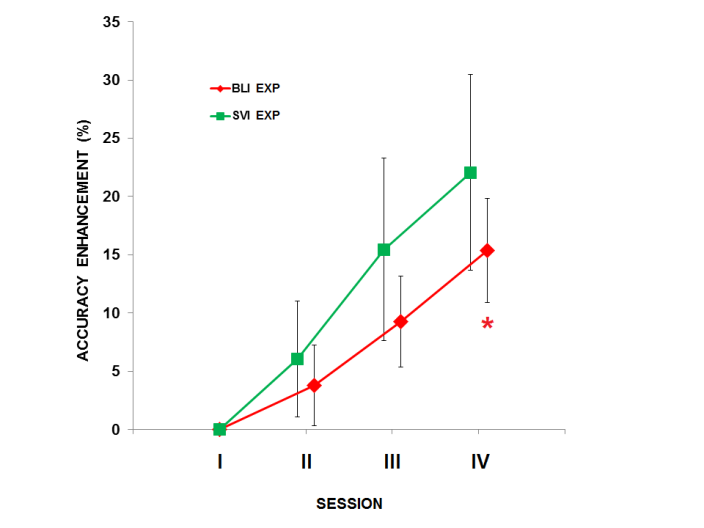
**

**Supplementary Figure 1. Normalized accuracy enhancement (SEM indicated as whiskers) across sessions in the distance discrimination task using the programmable tactile display. Color coded asterisk indicates higher accuracy compared to the baseline. *, *p*FDR-corrected < .05.**

**Raw accuracy: not all mistakes are equal**

To better assess training effects, we next refined our analysis of errors to consider by *how much* the participant was wrong, when giving an incorrect response. Our rationale was that, in principle, confusing the shortest distance pair with a slightly longer distance pair is very different than confusing it with a much longer distance pair. In the former case, the participant simply might have had a difficulty in discriminating hard-to-discriminate pairs, while in the latter case the participant might have misunderstood the concept of ‘short’ or ‘long’ distance. Recall that if the BlindPAD displayed N symbols, the number of possible pairs among which a choice has to be made is 0.5*N*(N-1), i.e. 3 pairs for 3 symbols, 6 pairs for 4 symbols, 10 pairs for 5 symbols, etc.

To assess this, we ordered the distances between squares for each trial in ascending order (i.e., from the closest pair to the farthest pair), assigning a number based on the ranked position (from 0 to N, where N is the number of possible pairs for that trial). We then computed the rank distance between a participant’s response and the correct response (Δ_accuracy_; i.e., we assigned a score = Δ_0_ for correct answer, a score = Δ_1,_ if the chosen distance was not the correct one but the second-best in ranking, and so on) for each trial of the experiment. We did it both for dmin and dmax. Practically, lower delta scores (e.g., Δ_1_) indicate less serious mistakes and higher delta scores (e.g., Δ_4_) more serious errors. We found most mistakes (91.1%) were Δ_1_ and Δ_2_ errors suggesting that, in general, participants had a good spatial awareness and understood the concepts of short and long distances. Having the majority of mistakes involving similar distances aligns with the idea, explored in the “normalized accuracy” section, that distances between squares may sometimes have been below the JND for the distance discrimination. The number of Δ_1_ errors was significantly higher than the number of Δ_2_ errors both for dmin and dmax (both *p* < 0.001).We then analyzed the distributions of Δ_1_ and Δ_2_ errors for dmin and dmax, as a function of the level of difficulty, or the total number of pairs among which a choice had to be made (Supplementary Figure 2).

For dmin, the level of difficulty affects the number of Δ_1_ (χ^2^=35.3, *p* < 0.001) but not Δ_2_ errors (χ^2^ = 3.5, *p* = 0.17). The percentage of Δ_1_ errors is higher in the 5-square (18%) condition compared to 3- (16%) and 4-square (10%) conditions (both *p*FDR-corrected < 0.05). Note that the increase in the number of Δ_1_ mistakes when going from the 3-square to the 6-square trial (3%) is small relative to the large difference in selecting the correct pair by random chance alone (e.g., for the 3-square test, random chance of picking the correct pair is 33% (only 3 possible pairs), whereas for the 6-square level, random chance of correctly picking the correct pair is 6.7% (15 possible pairs). The 5- and 6-square conditions did not differ in the number of Δ_1_ mistakes (*p* = 0.40), nor did the 3- and 4-square conditions (*p* = 0.055). Similarly, for dmax, the level of difficulty affects the number of Δ_1_ (χ^2^=8.48, *p* = 0.01) but not of Δ_2_ errors (χ^2^ = 2.13, *p* = 0.34). The percentage of Δ_1_ errors is higher in the 3-square (28%) condition compared to the 4- (23%) and 6-square condition (15%; both *p*FDR-corrected = 0.086). Similarly, the percentage of Δ_1_ errors is higher in the 4-square than in the 6-square condition (*p*FDR-corrected = 0.006). This pattern of decreasing errors with increasing level of difficulty presumably reflects the fact that level of difficulty was correlated with training session. Hence, learning across the training seems to counterbalance the effect of increased level of difficulty of the task.


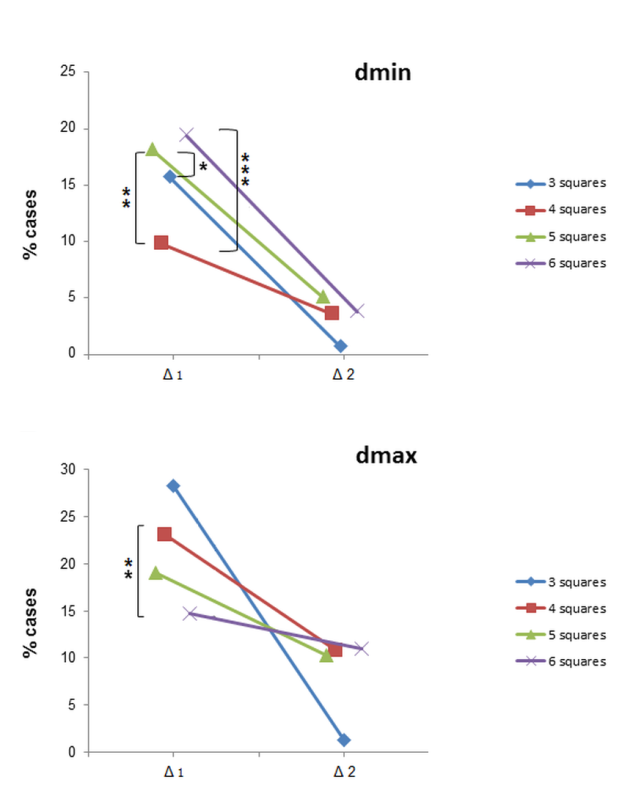


**Supplementary Figure 2. Percentage of Δ_1_ and Δ_2_ errors as a function of the level of difficulty for dmin (upper panel) and dmax (lower panel). *, *p*FDR-corrected < 0.05; **, *p*FDR-corrected < 0.01; ***, *p*FDR-corrected < 0.001.**

**Intra-session and inter-session effects**

**Accuracy**

As we observed a learning effect across sessions, we attempted to investigate whether the learning took place mainly within a session or in the inter-session interval (1 week).

Supplementary Figure 3 shows how accuracy varied at the intra-session level. We cannot observe an accuracy enhancement within a session. The average accuracy of the first three trials and the last three trials are not statistically different neither for dmin (Z = 176, p = .11) nor for dmax (Z = 840, p = .70).


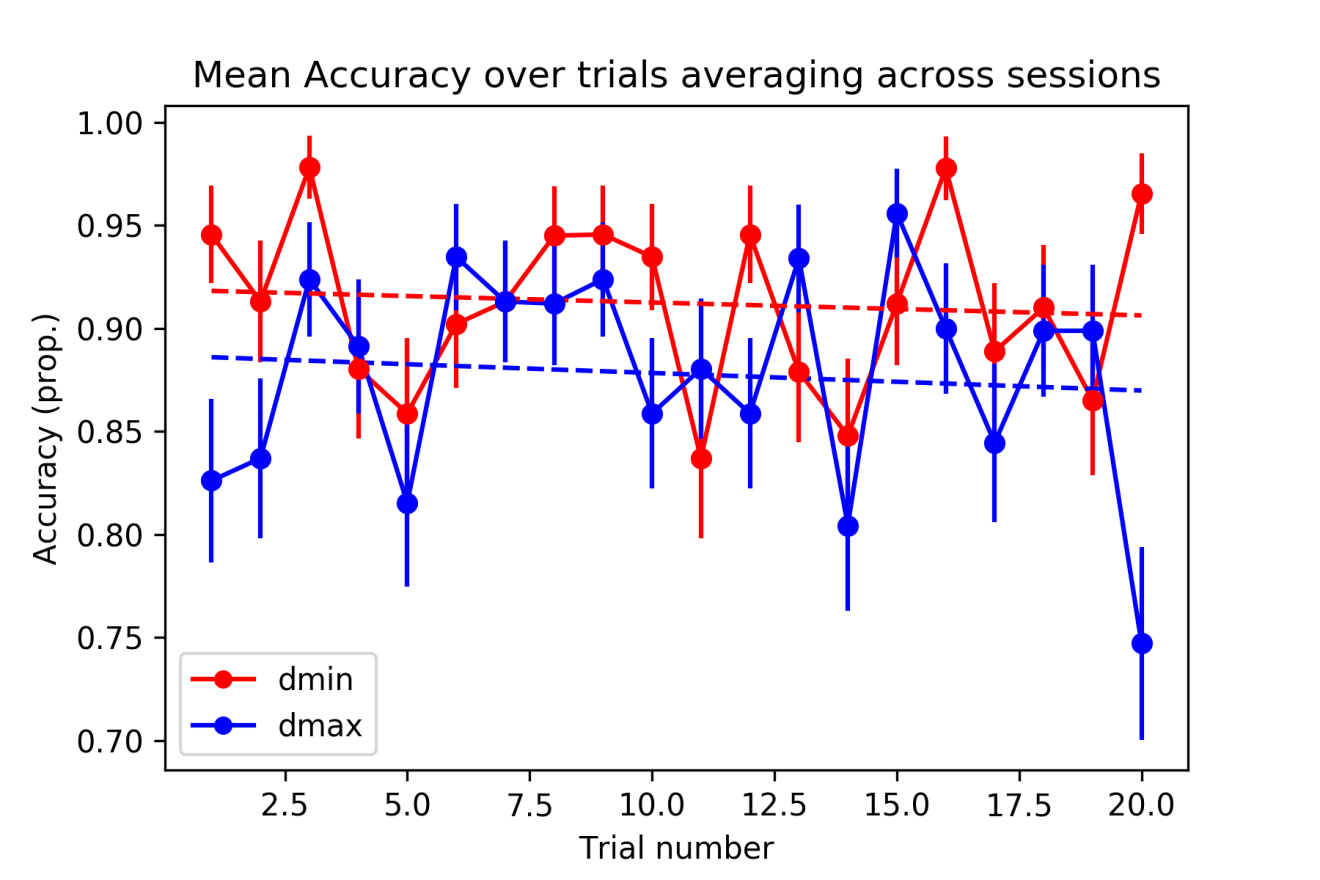


**Supplementary Figure 3. Proportion of accuracy (SEM indicated) for each trial after averaging for session and level of difficulty. Linear regression lines are plotted.**

As for the analysis of the inter-session learning effect, we compared the average accuracy of the threshold sessions to the average accuracy of the first and last 5 trials of the previous training session using the same level of difficulty. As for dmin, threshold accuracy (0.95) and the accuracy of the first (0.94) and last 5 trials (0.92) of the previous session are very similar (both p’s > .42). Also in the case of dmax, threshold accuracy (0.90) and the accuracy of the first (0.90) and last 5 trials (0.85) of the previous session are similar (both p’s > .23). Certainly, these statistics might be limited by the small sample sizes.

**Response Time**

Also in case of RTs, we verified the intra- and inter-session effects. Supplementary Figure 4 shows RTs trend at the intra-session level.


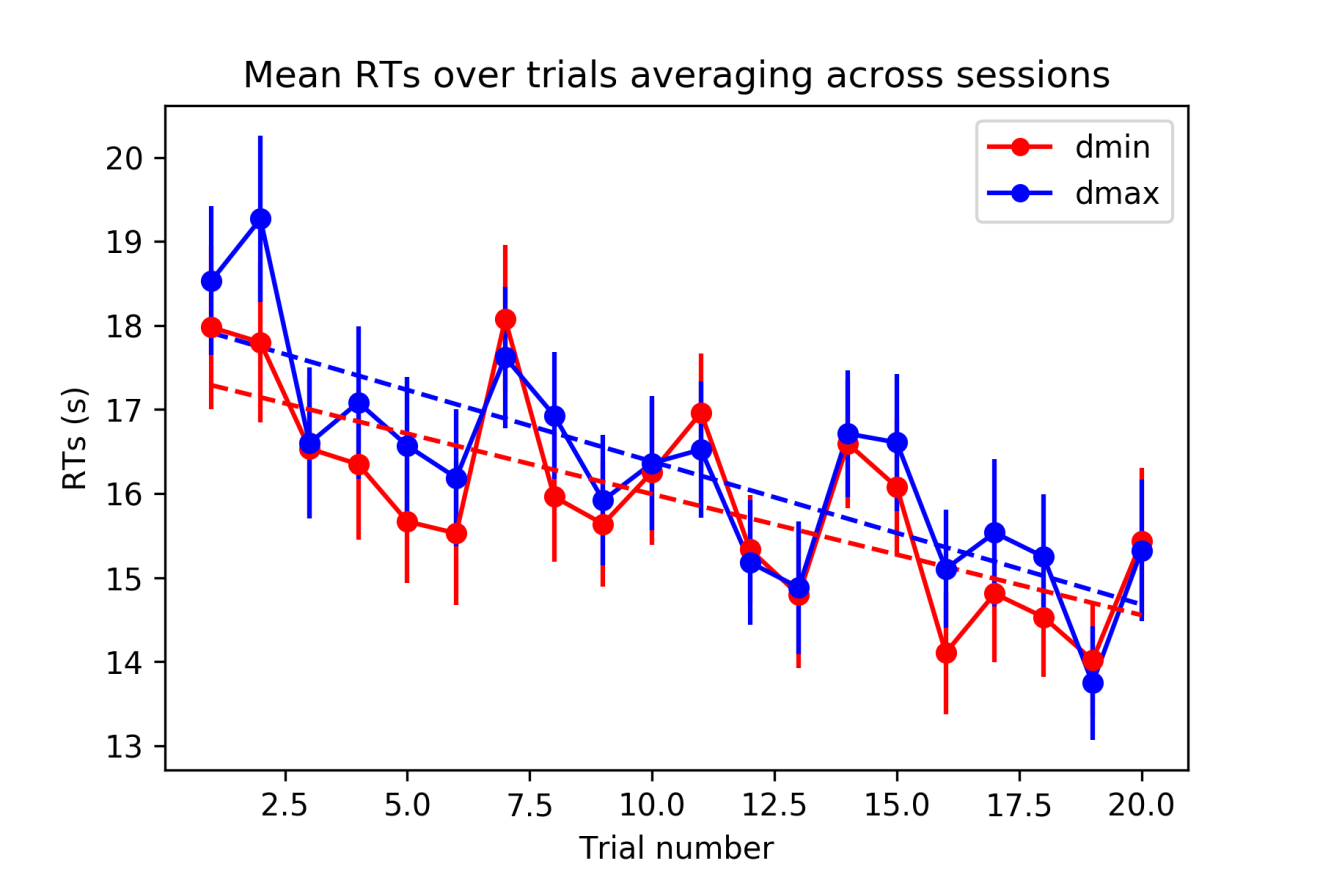


**Supplementary Figure 4. Response times in seconds (SEM indicated) for each trial after averaging for session and level of difficulty. Linear regression lines are plotted.**

A Friedman ANOVA showed a significant effect of the trial on response speed (χ^2^ = 66.98; p < .00001). The mean response of the first three trials (17.5 s) and the mean response of the last three trials (14.75 s) were indeed significantly different (Z = 2.91, p = .003). RTs in the last three trials were 15% faster than responses to the first three trials.

As for the inter-session effects, we repeated for RTs the analyses we did for accuracy. Results show that the mean RT of the threshold session is not different than the first 5 trials of the previous session both for dmin and dmax (both *p*’s > .55). On the contrary, the last 5 trials of a session tend to be faster than the 5 trials of the subsequent threshold session, especially for dmax (15.16 s vs. 17.34 s, p = .0462).
